# Practical Management of Epileptic Seizures and Status Epilepticus in Adult Palliative Care Patients

**DOI:** 10.3389/fneur.2018.00595

**Published:** 2018-08-02

**Authors:** Wenke Grönheit, Stoyan Popkirov, Tim Wehner, Uwe Schlegel, Jörg Wellmer

**Affiliations:** ^1^Ruhr-Epileptology, Department of Neurology, University Hospital Bochum, Bochum, Germany; ^2^Department of Neurology, University Hospital Bochum, Bochum, Germany

**Keywords:** palliative care, epilepsy, epileptic seizures, end of life, status epilepticus, non-convulsive status

## Abstract

In terminally ill patients, paroxysmal or episodic changes of consciousness, movements and behavior are frequent. Due to ambiguous appearance, the correct diagnosis of epileptic seizures (ES) and non-epileptic events (NEE) is often difficult. Treatment is frequently complicated by the underlying condition, and an approach indicated in healthier patients may not always be appropriate in the palliative care setting. This article provides recommendations for diagnosis of ES and NEE and treatment options for ES in adult palliative care patients, including aspects of alternative administration routes for antiepileptic drugs such as intranasal, subcutaneous, or rectal application.

## Introduction

Patients may require palliative care for several conditions. These are not only end stage systemic cancer including primary brain tumors and cerebral metastases, but also ischemic stroke, intracerebral hemorrhage, neurodegenerative diseases, and non-neurological conditions such as terminal liver, kidney, or respiratory failure. Many of these carry an increased risk for epilepsy and epileptic seizures (ES) or non-epileptic events (NEE, defined as paroxysmal or episodic changes of consciousness, movement and/or behavior) (1–4).

Both ES and NEE present an important challenge in the already complex interaction with palliative patients. Differential diagnosis and treatment can be difficult. Events are not always witnessed by professionals and even if so, focal seizures with reduced awareness and automatisms can be mistaken for delirium or agitation (5). If patients are found in a state of impaired consciousness, several reasons are possible, too, such as a postictal state, non-convulsive status epilepticus (NCSE), dehydration, metabolic dysfunction, or new intracerebral pathology such as ischemic stroke or hemorrhage. In elderly patients who are increasingly represented among palliative patients, event duration as a distinguishing feature is less useful than in young patients due to longer lasting postictal periods of impaired consciousness or focal neurological deficits (4, 6). The ability of patients to report subjective symptoms which might facilitate differentiation can be reduced in palliative patients.

Treatment with antiepileptic drugs (AEDs) including benzodiazepines requires careful consideration in palliative patients since they may have prolonged sedative effect. This can even obscure the diagnosis (7, 8). Nevertheless, if patients are confirmed to have epilepsy, anticonvulsive treatment is usually indicated, however, in palliative patients in particular the burden of side effects has to be balanced against the benefit of reduction of seizure frequency and severity (9). In palliative care side effects can be especially strong due to drug-drug interactions, impaired metabolism and systemic comorbidities (9–11). Cognitive or sedative side effects can rob palliative patient's remaining autonomy and negatively affect quality of life. Reliable routes of AED administration (which need to maintain constant blood level) is a particular challenge in patients with dysphagia and/or impaired consciousness.

The following sections provide recommendations for the differential diagnosis of ES and NEE and for treatment of ES in patients under palliative conditions.

## Diagnostics

### Semiology

Seizure semiology, the description of ictal signs and symptoms, is the most important diagnostic tool in epileptology. Characteristic semiological elements and their sequence can be used to ascertain the epileptic etiology of seizures (or NEE in turn) and to guide further diagnostic and treatment (12).

Since studies on seizure semiology in the palliative care setting are lacking, findings in elderly patients may be a helpful approximation. In a prospective study comparing young and elderly epilepsy patients, Stefan et al. found less clonic elements and a higher proportion of non-convulsive status epilepticus in elderly patients. In the light of more differential diagnoses, this leads to frequent misdiagnosis in particular in first manifestations of seizures (4, 13, 14).

Sheth et al. described that in elderly patients with ictal confusion the correct diagnosis was made only late (31 ± 30 h, range: 1–140) (14). Patients often appeared bewildered, had impaired attention and concentration, or had impairment of goal-directed action. Speech was reduced to simple semiautomatic phrases or gestures. Subtle ictal manifestations included a subtle gaze preference and low amplitude fragmentary myoclonic jerks, typically in the face, eyelids, or hands, and at times associated with hand automatisms.

Studies assessing symptoms in palliative care patients tend to report a relatively small prevalence of ES, but impaired consciousness of unspecified cause is very common with occurrences in up to 90% of cases (15). This discrepancy might indicate a high number of unrecognized seizures. Therefore, ictal or post-ictal states should be considered an important differential diagnosis of unexplained drowsiness.

As patient's descriptions may have limitations owing to their level of consciousness, the observer's description is frequently the only available source of information for physicians (16). Subtle semiologies are often not recognized and therefore remain unreported. Witnesses of seizures may use misleading descriptions of what they saw (17–19). And in palliative care patient's symptoms may often appear less “textbook-like” than in healthier patients. Therefore, every attempt should be made to get as close as possible to the actual semiology of the event.

Beniczky et al. reported that accuracy in diagnosis is higher when resulting from a dialogue between physician and patients or witnesses, respectively, compared to when symptoms are only reported (20). Recording characteristic episodes by smartphone videos is a valuable aid, too. To document the level of consciousness patients should be addressed verbally in the video sequence as recommended for video-EEG monitoring (21).

### Technical examination

Ideally, in palliative patients the diagnosis will be made at the patient's place of care without hospital admission. Yet, in some instances electroencephalography (EEG) may be required to make the correct diagnosis. In particular the diagnosis of NCSE is facilitated by EEG (22–24). EEG patterns of NCSE can be highly variable and sometimes difficult to distinguish from encephalopathy, and clinically suspected NCSE without typical EEG pattern are common (25). Sometimes only the combination of EEG and time limited treatment trial (see below) clarifies the situation. Since NCSE can fluctuate prolonged EEG recording can be necessary (26).

The diagnostic value of short term (routine) EEG in palliative care is difficult to rate, because EEG in elderly patients (and probably in palliative patients, too) rarely shows normal findings, instead focal slowing with or without epileptic discharges is frequent (27). Absence of epileptic discharges, on the other hand, does not exclude epilepsy.

If EEG shows clear epileptiform discharges, the risk for seizure recurrence is considerably higher and the diagnosis of epilepsy might be made after only one seizure [practical definition of epilepsy; (28)]. However, EEG readers should be aware that the combination of a NEE and an overinterpreted EEG (e.g., mistaking sleep signs as epileptiform discharges) may lead to a misdiagnosis and avoidable use of AED. Therefore, before application of EEG the clinical assessment of the event in question is of paramount importance.

MRI is the most sensitive imaging method regarding epileptogenic lesions (29). In palliative patients with new-onset seizures, imaging could in fact reveal brain metastases, meningeosis, brain tumor progression, or stroke or verify metastases of already diagnosed systemic cancer (10). Yet, in patients in palliative setting MRI only needs to be performed if recognition of brain pathology had therapeutic consequences. Computed tomography (CT) may be less sensitive for the above pathologies, but will reveal most of the relevant reasons, like gross tumors or brain edema, and is much less time consuming and less of a burden for the patients. Therefore it might be more adequate in these patients.

Laboratory testing has some relevance in the differential diagnosis of ES and NEE. The focus is, however, more on detecting conditions that mimic ES such as dysglycaemia, electrolyte imbalance, hyperammonemia, anemia rather than on verifying epileptic seizures. Postictal creatine kinase (CK) elevation in serum is helpful only after generalized tonic clonic seizures (GTCS), but in palliative patients the reliability of CK elevation after GTCS is unknown due to often reduced muscle mass. CK in general can be substantially lower in the elderly (30). Serum prolactin measurement has no use in the palliative care setting.

Measuring AED blood levels is useful in cases when drug intoxication is part of the differential diagnosis [e.g., valproic acid (VPA) intoxication vs. NCSE], but this is rare and regular blood sampling should be avoided in the palliative setting.

In case of doubt concerning semiology based differential diagnosis and the application of technical examinations it might be helpful to get the opinion of an epileptologist though undoubtedly, in some cases a precise diagnosis might remain impossible.

### Antiepileptic therapy

Seizures interfering with Quality of life (QoL) should be treated, though anticonvulsive treatment *per se* should not impair QoL. We propose the following principles to guide anticonvulsive treatment in epilepsy patients in the palliative care setting:

#### Respecting patient resources and wishes

Brom et al. found 93% of the patients in the palliative setting prefer to share responsibility with their physician in clinical decision making (31). Because cognitive problems may hamper communication and thus shared decision-making as brain diseases progress, advanced planning is crucial (15).

#### Target levels of QoL

Treatment regimes should be chosen to protect or improve activities of everyday life that are important to the individual patient (e.g., not accepting daytime sleepiness for complete seizure freedom).

#### Considering the current and future requirements of therapy

Avoiding interactions with medications used to control other symptoms (e.g., steroids, palliative chemotherapy) (10) and between AED themselves.

#### Ensuring practicality

Choosing application forms easily applicable by the patients themselves, family and caregivers. Since disease progress and/or symptom fluctuations can make swallowing of tablets or capsules difficult temporarily or permanently, treatment plans should enable flexibility in this respect to avoid acute withdrawals.

The threshold for seizure medication cessation in the end of life-setting should vary according to the kind of pre-existing epilepsy (low threshold for single post-stroke seizure a year ago vs. high threshold for long-standing structural epilepsy.) However, this is a highly individual decision in all cases, even if comprehensive medical records are available and may be approached using a shared decision making process involving the patient and caregivers.

### Acute management of seizures and convulsive status epilepticus

As most epileptic seizures are self-terminating, there is no need to apply acute anticonvulsive treatment during or after every seizure. This accounts for a first in lifetime seizure in palliative patients, too. The rationale behind this is that, in addition to postictally impaired consciousness and a potential acute seizure cause, AED, in particular benzodiazepines, may impair consciousness—sometimes for days. Acute administration of AED in palliative patients should thus be restricted to status epilepticus (SE) or series of seizures.

While the current definition of status epilepticus applies to palliative epilepsy patients (32), not all treatment recommendations can be transferred.

Patients should be acutely treated when a generalized seizure lasts longer than 5 min (so-called continuous seizure activity, or early SE) or two or more seizures occur without regaining pre-ictal level of consciousness in between events (32, 33). Choice of treatment of SE will depend on the patient′s location: hospital, hospice, or home care.

In either setting, the first step of treatment (0–10 min) is administration of benzodiazepines. Due to its pharmacokinetic characteristics (e.g., long antiepileptic effect conditional on slow redistribution in the body fat), lorazepam is often preferred as initial treatment of SE (33). Alternatively, midazolam has been proven equally effective (34).

Formal recommendations for starting doses in a palliative situation do not exist, but application of lower doses of benzodiazepines, if necessary repeated, may be preferable over the initial application of the maximum recommended dose. Future research should address if in the specific setting of palliative patients, the initial application of non-sedating, easily applicable fast acting AED such as levetiracetam (LEV) or brivaracetam (BRV) may prove advantageous.

#### Hospital setting

Intravenous status therapy and even intensive care treatment can be reasonable acknowledging that early beginning of treatment increases the chance of seizure termination (35). Therefore, first steps of in-hospital treatment of SE in the palliative situation can be adopted from the general treatment recommendations for SE (Table 1). In established SE (10–60 min), intravenous drugs [e.g., phenytoin/fosphenytoin, valproate (VPA), LEV, phenobarbital (PB)] are most commonly used, although there is no class I evidence for choosing one over the other (33). Among those VPA, LEV, and lastly additional lacosamide (LCM) seem to be effective and safe alternatives (33).

**Table 1:** Administration routes and characteristics of antiepileptic drugs relevant for palliative care.

**AED**	**Daily Dose**	**Special consideration for palliative care**	**IV**	**Liquid solution**	**Suspension**	**Tablet**
Brivaracetam (BRV)	50–200 mg	Mild CYP3A4 metabolism. Probably no clinical relevant interactions	+	+	–	+
Carbamazepine (CBZ)	600–2000 mg	Dizziness, nausea, ataxia Effective for neuralgic pain (200-400 mg/d), decreases: VPA, TPM, LTG, neuroleptics, antimycotic agents, antidepressant drugs, steroid level Increases: diazepam level and effective CBZ- Metabolite Is decreased by: PHT Is increased by: Theophyllin, Cisplatin	–	+	+	+
Eslicarbazepine (ESL)	800–1600 mg (max. 1200 mg when combined with other AED)	Dizziness, gait disturbance, ataxia, hyponatremia Is decreased by: PHT, CBZ Increases: PHT	–	–	–	+
Gabapentin (GBP)	900–3000 mg	Sedation (especially in combination with opioids), therapy of neuropathic pain (900 mg/d) Is increased by: morphine	–	+	+	+
Lacosamide (LCM)	100–600 mg (max. 400 mg when combined with other AEDs)	Dizziness No relevant interactions	+	+	+	+
Lamotrigine (LTG)	100–300 mg	Tremor, sedation (rare), sleep disturbance„ mood stabilizing effect. Very slow titration necessary Is decreased by: CBZ, PHT Is increased by: VPA	–	–	+	+
Levetiracetam (LEV)	1000–3000 mg (−4000 mg off-label) mg	Sedation (rare), psychiatric side effects No relevant interactions	+	+	+	+
Oxcarbazepine (OXC)	900–2400 mg	Dizziness, nausea, ataxia (less often when the slow release form is used), hyponatraemia	–	+	+	+
Perampanel (PER)	4–12 mg	Dizziness, somnolence Is decreased by: CBZ, OXC, TPM Decreases: CBZ, OXC, VPA	–	–	–	+
Phenytoin (PHT)	200–350 mg	Dizziness, allergy. Potentially complicated titration Decreases: steroid level	+	–	+	+
Pregabalin (PGB)	150–600 mg	Sedation. No relevant interactions Anxiolytic effect.	–	+	+	+
Topiramate (TPM)	50–200 mg	Sedation, fatigue, lack of appetite, weight loss, paraesthesia, speech disturbances No relevant interactions	–	–	+	+
Valproate (VPA)	1200–2400 mg	Tremor, encephalopathy, mood stabilizing effect. Enzyme inhibition (leading e.g., to increased toxicity of chemotherapy).	+	+	+	+

The idea of palliative care is to balance noninvasive treatment and avoiding delays in optimal (but invasive) therapies (e.g., deduced from ICU treatment of patients with cancer: “unlimited ICU support for a limited time period”), respecting the patient's wishes (36). Knowing refractory and super-refractory SE are treated with anesthetics with a markedly lower success rate and a high morbidity and mortality (33, 37), it seems difficult to apply these principles to the palliative care setting. In some cases “palliative sedation” using benzodiazepines (or alternatively propofol) might alleviate symptoms even if epileptic activity persists.

#### Hospice/home care setting

In SE in children, intranasal or buccal midazolam or lorazepam or IM- midazolam have been found to be at least equally effective as the IV or rectal form (34, 38–43). Although data in adults are limited (34, 44) the differences among various non-intravenous routes are likely to be small. The non-IV application forms of benzodiazepines can be administered by family members or carers and are thus a valuable tool in the therapy of SE in a hospice or home care setting where the IV-route is typically not available (Table 1) (45).

In patients with recurrent episodes of prolonged seizures or SE, benzodiazepines should be administered as early as possible to shorten the seizure.

Addressing practical issues, lorazepam has been shown to be stable at room temperature (20–25°C) for at least 0.8 months (46). Although it showed some chemical degradation after 60 days, the concentration of the active metabolite remained at acceptable levels. Midazolam was found to be stable for 60 days (47). Therefor these medications can be kept at the patient's bedside to allow fast administration if necessary. This is of particular relevance as seizure frequency increases in the last weeks of life (48).

### Non convulsive status epilepticus (NCSE)

Samala et al. noted that in terminally ill patients, successful treatment of NCSE can restore the ability to communicate, facilitate goals of care discussion and positively impact QoL (49). Lorenzl et al. shared this opinion in lining out that the most notable effect of treating NCSE was regaining the ability to communicate (50). Because NCSE is potentially responsive to therapy, treatment should be considered in all patients and started as soon as (50) possible.

As outlined above, diagnosing NCSE or SE in the palliative care setting can be challenging. Drislane et al proposed to include the response to anticonvulsant as a diagnostic criteria, in addition to the semiological and EEG features discussed above (51). Therefore, probatory therapy seems to be a reasonable approach in the palliative setting, given that a prolonged confusional state following a GTCS might in fact be due to ongoing NCSE and a history of epilepsy is a risk factor for this condition as well.

The initial treatment of NCSE does not differ from the approach outlined for GCSE outlined above. The first step should be the administration of a benzodiazepine followed by LEV, LCM, or VPA, if necessary. In a palliative situation, these drugs may be administered orally, sublingually, via an NG or PEG tube, or subcutaneously [(50), Table 2].

**Table 2:** Proposal for (convulsive) SE treatment in palliative care [partially taken from (33)].

		**Hospital**	**Hospice/home care**	**Outcome**
Stage 1 5–10 minutes	Early phase Premonitory SE, Impending SE	Lorazepam IV 0.05 mg/kg max. 2 mg/minute, if necessary repeat after 5 minutes	Midazolam buccal or intranasal 0.2 mg/kg (5–10 mg) or Lorazepam buccal or intranasal 0.05 mg/kg or 10 mg IM-midazolam Repeat if necessary	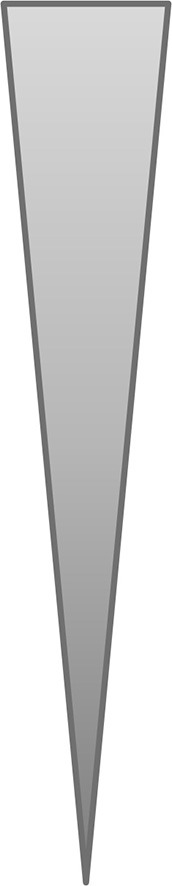
Stage 2 10–30 minutes	Established SE	Levetiracetam 30-60 mg/kg IV max. 500 mg/minute, if necessary repeat after 10 minutes and/or additional lacosamide 5 mg/kg IV in 15 minutes *Alternative stage 2:* Valproate 20-30 mg/kg IV max. 10 mg/kg/minute, if necessary repeat after 10 minutes	*In absence of IV route:* 1000–2000 mg levetiracetam in 100 ml 0.9% sodium chloride over 30 minutes SQ if necessary additional: 200 mg lacosamide over 20 minutes SQ Repeat if necessary, or repeat benzodiazepine administration	
Stage 3 30–60 minutes	Refractory SE: SE, that continues despite stage I/II treatment, subtle SE, stuporous SE	midazolam bolus 0.2 mg/kg IV, continuously 0.1–0.5 mg/kg/h or propofol bolus 2 mg/kg IV, continuously 4–10 mg/kg/h	consider palliative sedation	
Stage 4 >24 h	Super refractory SE: SE, that continues despite treatment with anesthetics >24 h	consider palliative sedation	consider palliative sedation	

### Application forms of AEDs

Dysphagia is a common symptom in neurological and oncological patients (15, 52). Independent of the underlying disease, swallowing might be affected by a reduced level of alertness, inattention, and muscular weakness. Therefore, it is helpful if an AED can be administered as an oral liquid, subcutaneously, or rectally.

#### Subcutaneous AED application

Subcutaneous (SQ) administration of LEV has been shown to be safe and effective as a continuous infusion via a syringe driver (250–4000 mg/d, dosage equal to prior oral route), or intermittent bolus diluted in 100 ml 0.9% sodium chloride every 12 h over 30 min. In 20 prospectively examined patients, 7 showed seizure activity under SQ administration, leading to an increase of the SQ dose or addition of a benzodiazepine (53). Rémi et al. identified 20 patients treated with SQ LEV without adverse reaction at the infusion. In 16 patients (80%), no further seizures were noticed or SE was terminated (54).

Moreover, Rémi et al. described one patient receiving SQ LCM. According to the former oral dosage, 200 mg of the undiluted LCM solution over 10 min SQ twice a day was administered and well tolerated. Serum levels were in the recommended range and their course comparable to oral administration (55).

#### Rectal AED application

The rational of rectal antiepileptic administration is avoidance of hepatic first pass effect due to rectal venous drain. Anderson et al. suggest rectal administration to be feasible for short term administration of carbamazepine (CBZ), lamotrigine (LTG), LEV, PB, topiramate (TPM), and VPA (56).

Birnbaum et al. found compressed LTG to be rectally absorbed and well tolerated in 12 healthy adults (57). Chewable dispersible LTG was tested likewise in 12 healthy adults but was not absorbed to the same extent compared to oral administration (58).

Conway et al. showed TPM to be absorbed to a similar extent as the oral dosage when administered rectally in 10 healthy adults (59).

Stockis et al. monitored pharmacokinetic data during targeted delivery of LEV to the colon (60). Systemic bioavailability after application in the ascending colon was comparable to oral administration. This suggests that LEV may be administered rectally (56).

VPA has been shown to be highly absorbed (80%) after rectal administration in healthy adults. Its peak serum concentration was ~30% lower and achieved 2.1 h later when compared to oral intake (3.1 vs. 1 h) (61). Multiple studies and case series demonstrate its clinical practicability and effectiveness (61–65).

It is important to note that many of these recommendations, although widely used, are off-label, and patients and caregivers should be informed accordingly.

## Conclusion

ES are a relevant clinical problem in a palliative care setting that may affect the patient's QoL. They thus require adequate supportive care and treatment. Timely recognition and adequate out of hospital management may prevent unnecessary hospital admissions for uncontrolled seizures (66). In line with the general philosophy of palliative care, seizures should be addressed like other symptoms that may cause discomfort or reduce QoL. The patient's wishes and needs should shape the anticonvulsant therapy as early as possible. Careful use of alternative AED administration routes can lead to a very individualized and practical therapy regime even in the last days of life. Seizure recognition, acute management, drug administration, and possible side effects are all areas where caregivers might benefit from education and training (3, 15).

What outstanding questions should be addressed by future research in this area? Randomized or controlled studies will be difficult to conduct in palliative care settings. Future research to improve seizure management should include pharmacokinetic studies on alternative administration routes combined with respective case series; descriptive studies on seizure semiology in the terminally ill; and studies on service provision regarding transdisciplinary communication.

## Author contributions

WG wrote the first draft of the manuscript. All authors (WG, SP, TW, US, and JW) revised the manuscript critically and approved the final version.

### Conflict of interest statement

WG received speaker's honoraria, travel, or accommodation payment or participation fee from UCB, Eisai and Bial. JW has received speaker's honoraria from UCB, Desitin, Eisai und Bial. US has received speaker's honoraria from GSK, Novartis, medac and has received consultancy fees from Roche. SP reports no conflicts of interest to this work. TW has received consultancy fees by Bial and speaker fees for participation at educational activities organized by Eisai, Pfizer and UCB.

## Abbreviations

ES: epileptic seizure
NEE: non-epileptic event
AED: Antiepileptic drugs
NCSE: non-convulsive status epilepticus
EEG: Electroencephalography
CT: Computed tomography
CK: Creatine Kinase
SE: status epilepticus
GTCS: generalized tonic clonic seizure
QoL: Quality of life
ICU: intensive care unit
SQ: subcutaneous
IV: intravenous
IM: intramuscular
LEV: levetiracetam
LTG: lamotrgin
BRV: brivaracetam
PB: phenobarbital
VPA: valproic acid
LCM: lacosamide
TPM: topiramate
CBZ: carbamazepine
NG: nasogastric
PEG: percutaneous endoscopic gastrostomy.
